# The Use of Tobacco by Boys

**Published:** 1882-11

**Authors:** 


### THE USE OF TOBACCO BY BOYS.

The use of tobacco by growing boys is so generally recognized as pernicious that it is extraordinary that more energetic measures are not urged upon those having the care of youth to prevent the habit. Already it has been prohibited in the United States Naval Academy, at Annapolis ; in the United States Military Academy, at West Point ; in the Phillips Exeter Academy, New Hampshire, and in various other enlightened educational institutions.

This was not the result of prejudice or hobbyism. If any set of men are free from these vices of learning, it is the naval surgeons, and it was especially from them, and particularly from Dr. A. L. Gihon, United States Navy, that this attack on the weed began. The indictment laid against it charged : That it leads to impaired nutrition of the nerve centers ; that it is a fertile cause of neuralgia, vertigo, and indigestion ; that it irritates the mouth and throat, and thus destroys the pur'ty of the voice ; that, by excitation of the optic nerve, it produces amaurosis and other defects of vision ; that it causes a tremulous hand and an intermittent pulse ; that one of its conspicuous effects is to develop irritability of the heart; that it retards the cell change on which the development of the adolescent depends.

This is a formidable bill of particulars, and yet each of these charges is preferred by the best modern authority, and, what is more, each is substantiated by an abundance of clinical evidence. Testimony is also adduced from the class records of schools and colleges, which indicate very positively that the effec' of tobacco on the mental facultles is deteiiorating. The best scholars are not tobacco users ; non-smokers take the highest rank in every grade ; and whether we look at the exceptionally brilliant students, or compare the average of those who use and those who refrain from tobacco, the result shows the same.—*Medical and Surgical Reporter.*

OPIUM kills 100,000 persons annually in China.

